# Announcement

**Published:** 1917-11

**Authors:** Alexander Lambert

**Affiliations:** Chief Surgeon of the American Red Cross in France


### The Medical Bulletin

N° 1 PARIS NOVEMBER 1917

### ANNOUNCEMENT

To " gaze humanely out upon human kind " and to act accordingly have always been the aims of the Red Cross. From the beginning of its existence it has endeavored to give help to the wounded of all nations. As the conception of this work has developed, so the breadth of its activities has enormously increased. From care in war it has extended its endeavors to the relief of sufferers from disasters and from epidemics of disease in time of peace.

As a further development of this humanitarian service, the American Red Cross, during the present great war, is endeavoring to extend aid to both military and civil populations alike. No one has ever questioned its right, even its duty, to care for the wounded soldier, or the sick soldier, or the one permanently disabled. The present commissioners of the Red Cross in France are prepared to go farther, for they believe that it is their duty to endeavor to assist, where their help is desired, in the scientific rese arch of the medical men caring for the American troops, that by such aid in scientific work the troops may receive more quickly the benefit of increased medical and surgical knowledge, both in the prevention of disease and in its treatment.

There is no question that when the organism of trench fever is found, and through the accurate knowledge of its life history a successful treatment preventing this disease is worked out, the health of the troops will be greatly increased, and the effectiveness of the armies enhanced. The discovery before the war of the spirochaete causing Weil's disease has solved the diagnosis of many baffling cases of fever. The discovery of the mechanism by which infection with the Bacillus aerogenes capsulatus causes gas gangrene has enabled the surgeons of all armies to save many a valuable life. This last discovery has been made since the war began, as the direct result of scientific endeavor under private support.

Such considerations show the value of this new activity of the

Red Cross, and bring it within the limits of the society's natural field of service. It is as important to help in protecting our troops against disease by methods of prophylaxis, as it is to relieve them in their sufferings resulting from the want of such means.

To this end the Red Cross desires to assist scientific work carried on in the American base hospitals or army laboratories in France. The society keenly appreciates that the best work can be done by those men who have the greatest clinical opportunities, and who are interested in the individual problems investigated. The scientific problems, therefore, should be met where there are the best opportunities, and the work be directed by any observer especially fitted for the investigation.

The following Research Committee has been appointed by the Commissioner of the American Red Cross in Europe, and under the supervision of this Committee, the research activities of the Red Cross will be carried on :

Capt. Walter B. Cannon, Chairman
Major Joseph A. Blake
Major G. W. Crile
Major H. Cushing
Col. M. W. Ireland
Major Alexander Lambert
Dr. James A. Miller
Major H. T. Nichols
Major Richard P. Strong
Dr. Homer Swift
Capt. Kenneth Taylor, Secretary
Dr. William C. White

(*All applications for information or assistance* should be made to the secretary of the Research Committee at 6, rue Piccini, Paris, XVI$^{e}$).

A central laboratory in Paris has been established under Capt. Taylor at the Red Cross Military Hospital, N$^{o}$ 2, at 6, rue Piccini, which will cooperate with the present laboratory there established under the Robert Walton Goelet Research Fund. It will be equipped as fully as possible, and it is hoped that workers from other military hospitals will utilize it for the completion of problems requiring special facilities.

It is hoped that assistance may be extended also to scientific workers of our allies. Many English and French surgeons have expressed their willingness to cooperate in the study of the scientific problems of the war.

To disseminate the knowledge thus obtained more effectively the Red Cross proposes : First, to encourage periodic meetings of the men concerned; Second, to make available the reports of the latest methods of treatment for war injuries and diseases by means of publication, and through a library established for this purpose.

In order to provide for meetings it seems advisable to found a research society to meet once a month in Paris, thus permitting men engaged in scientific investigations to exchange views. To review the medical literature, a journal, the first number of which is the present issue, has been started : it will contain abstracts of papers read at the monthly meetings of the society, and also of articles appearing in the French, English, and American journals. It is published under the direction of the Bureau of Medical and Surgical Information, which is a department of the Medical Bureau of the American Red Cross in France. As a further means of disseminating recent medical knowledge, a library of current medical journals is being established at the office of the Bulletin, 6, rue Piccini.

It is the policy of the Medical Bulletin to publish no original articles, for fear of preventing the publication of such articles in standard medical journals which would assure them a more permanent record. The Bulletin, however, as the official organ of the Research Society, will include reviews of the papers read at the regular meetings. It will be issued monthly, and will be available for the physicians and surgeons with the American Army in France, and for those of our allies who may find it helpful.

Alexander Lambert,
Chief Surgeon
of the American Red Cross in France

